# Antibiotic-loaded bone cement fixation technique combined with bilateral pectoralis major muscle flaps tension-free management for sternal infection after midline sternotomy

**DOI:** 10.1186/s13019-024-02749-0

**Published:** 2024-05-14

**Authors:** Xia Jiang, Yong Xu, Mingqiu Li, Guoqing Jiao, Xiaosong Rong, Fanyu Bu

**Affiliations:** 1 Department of Cardiovascular Surgery, the Affiliated Wuxi People’s Hospital of Nanjing Medical University, Wuxi People’s Hospital, Wuxi Medical Center, Nanjing Medical University, No.299 Qingyang Road, Wuxi, Jiang Su Province 214203 China; 2 Department of Chronic Wound, Wuxi Ninth People’s Hospital affiliated to Soochow University, No.999 Liangqing Road, Wuxi, Jiang su Province 214062 China

**Keywords:** Deep sternal wound infection, Antibiotic-loaded bone cement, Bilateral pectoralis major muscle flaps, Tension-free

## Abstract

**Introduction:**

Deep sternal wound infection (DSWI) after midline sternotomy of cardiac surgery is a challenging complication that affects the outcome of surgery. This study aims to assess the clinical effectiveness of the antibiotic-loaded bone cement fixation technique combined with bilateral pectoralis major muscle flaps tension-free management in the treatment of DSWI.

**Methods:**

We retrospectively analyzed 5 patients with DSWI who underwent antibiotic-loaded bone cement combined with bilateral pectoralis major muscle flaps for chest wall reconstruction after sternotomy for cardiac surgery in a tertiary hospital in China from January 2020 to December 2021. The clinical and follow-up data were retrospectively analyzed.

**Results:**

All patients had no perioperative mortalities, no postoperative complications, 100% wound healing, and an average hospital stay length of 24 days. The follow-up periods were from 6 to 35 months (mean 19.6 months). None of the cases showed wound problems after initial reconstruction using antibiotic-loaded bone cement combined with bilateral pectoralis major muscle flaps.

**Conclusions:**

We report our successful treatment of DSWI, using antibiotic-loaded bone cement fixation technique combined with bilateral pectoralis major muscle flaps tension-free management. The clinical and follow-up results are favorable.

## Introduction

Deep sternal wound infection (DSWI) is an infrequent but life-threatening infection complicating major heart surgery that affects between 1 and 3% of patients undergoing cardiac surgery [1–3]. Whilst advances in treatments, DSWI causes longer hospital stays and increased cost, with associated mortality ranging from 14 to 47% [4–6]. Conventional therapy of DSWI provides antibiotics, surgical debridement, resuturing or vacuum sealing drainage (VSD). Unfortunately, in some cases these methods are insufficient [7, 8]. Some patients do not respond and develop large defects and chronic wounds, requiring flap reconstruction and therefore are referred to plastic surgeons [9, 10]. There are various local flaps used in the reconstruction of sternal defects, of which the pectoralis major muscle flap is considered a workhorse flap [11, 12].

Poly(methyl methacrylate) PMMA bone cement is comprised of powder and liquid-based components: the powder components are pre-polymerized PMMA beads, an initiator (benzoyl peroxide) and a radiopaque element (BaSO4 or ZrO2 particles) [13]. It is one of the most widely used thermoplastic acrylics, has been used for defect filling, fracture stabilization, and implant fixation [14–16]. The addition of antibiotics to PMMA bone cement was successfully introduced as a means to treat infection around prosthetic joints in 1970 [17]. Antibiotic-loaded bone cement (ALBC) acts as a carrier for the sustained release of antibiotics, which is nontoxic and does not elicit a meaningful immune response, has been used in clinic for the infection prevention and treatment [18, 19]. Bilateral pectoralis major myocutaneous flap (BPMMF) is most commonly used to reconstruct large sternal defects, which may be considered a first-line treatment for mediastinal infections [20, 21]. An important limitation of the pectoralis major flap is represented by limited coverage of the inferior third of the sternotomy wound [22]. Despite surgical advances in cardiac surgery and improvements in perioperative care, DSWI remains a devastating post-operative complication.

Currently, the treatment of DSWI has no gold-standard approach, sternal dehiscence and mediastinitis still continue to be an important problem that is difficult to treat. We developed a new treatment option for DSWI managed by ALBC combined with BPMMFs. The purpose of this paper is to outline the advantages of this technique and evaluate the mid-term results of this procedure.

## Methods

### Clinical data

We retrospectively reported 5 patients who received ALBC combined with BPMMFs for DSWI after cardiac surgery between January 2020 to December 2021. For Patient 2, classical management included multiple debridements, VSD and antibiotics failed. Facing this treatment failure, a combined approach with ALBC and bilateral pectoralis major muscle flaps tension-free coverage was elected. This strategy was the primary method used for the other four patients once they were diagnosed as DSWI. Patients after midline sternotomy of cardiac surgery who were diagnosed with DSWI using other treatment approaches were excluded. General data of patients were summarized in (Table 1). All patients with DSWI were managed with the same protocol. The informed consent forms were obtained from all patients.

**Table 1:** General patient data

Patient number, sex and age	BMI	LVEF (%)	Original procedure	Re-sternotomy	LITA	Comorbidities
No.1, male, 72 years	27.3	57	CABG	Yes	Yes	HBP, circulatory arrest COPD, smoking
No.2, female, 74 years	24.2	61	MVR+TVP+CABG	No	No	HBP, DM, COPD
No.3, male, 65 years	25.8	62	Bentall+Total arch replacement	Yes	No	HBP, smoking
No.4, female, 70 years	22.3	73	MVP+TVP	No	No	HBP, DM
No.5, male, 67 years	24.5	48	AVR+MVR	No	No	HBP, smoking

*Abbreviations* CABG, Coronary artery bypass graft; AVR, Aortic valve replacement; MVR, Mitral valve replacement; MVP, mitral valvuloplasty; TVP, tricuspid valvuloplasty; HBP, Hight blood pressure; DM, Diabetes mellitus; COPD, Chronic obstructive pulmonary disease; LVEF, Left ventricular ejection fraction; LITA, left internal thoracic artery

### The Centers for Disease Control and Prevention (CDC) defines DSWI as having one of the following criteria

1. An organism isolated from culture of mediastinal tissue/ fluid;
2. Evidence of mediastinitis seen intraoperatively;
3. Presence of chest pain, sternal instability, or fever (> 38°C), and purulent drainage from the mediastinum or isolation of organism present in a blood culture or from the mediastinal area [23].

### Surgical procedure

**Fig. 1:**
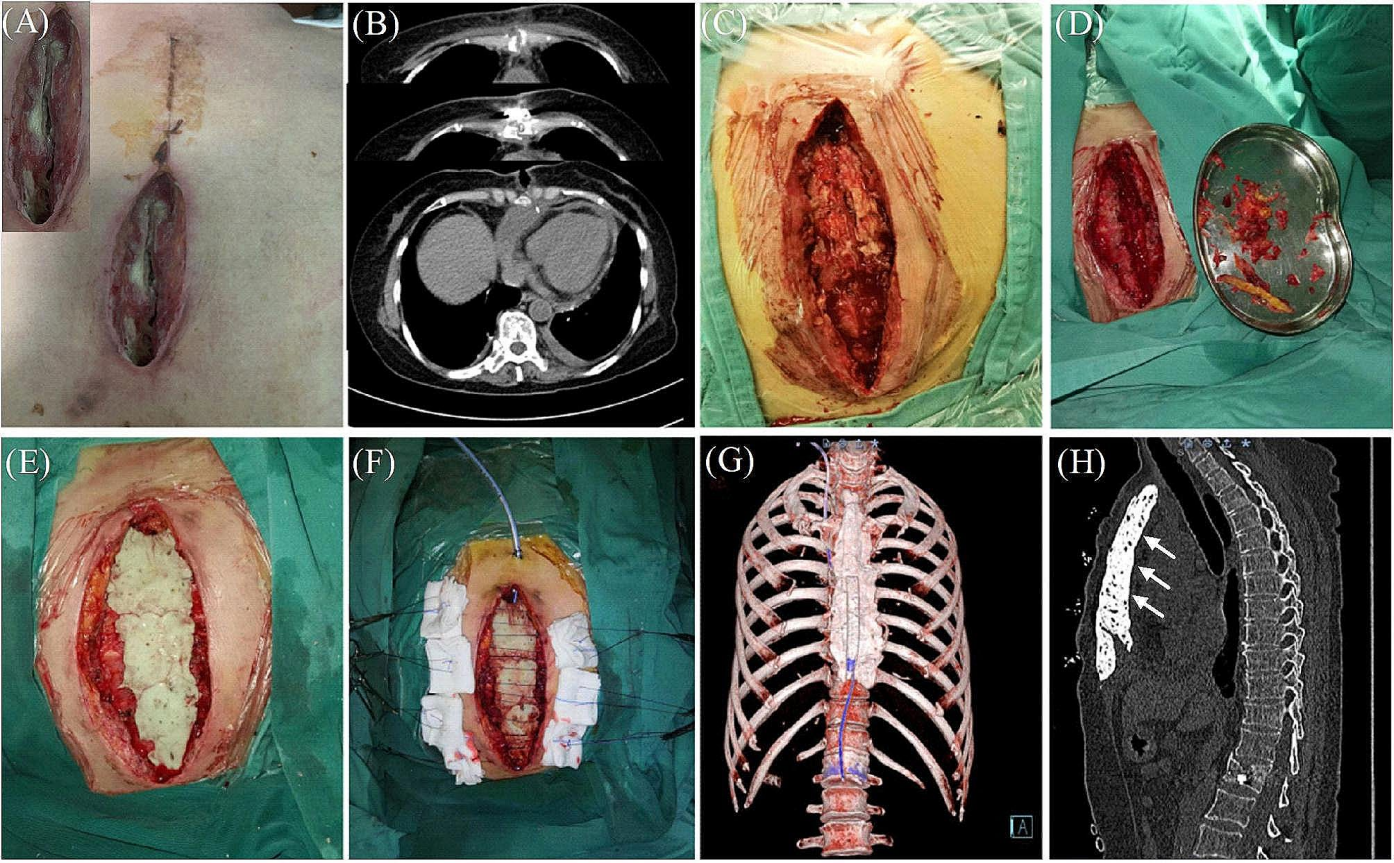
(**A**) Post-sternotomy sternal dehiscence and necrosis. (**B**) Preoperative thoracic computed tomography showing sternal dehiscence. (**C**) Open the wound completely. (**D**) The wound after debridement. (**E**) Antibiotic-loaded bone cement was sufficient to cover the whole length of sternum defect. (**F**) bilateral pectoralis major muscle flaps tension-free suture. (**G**) Three-dimensional chest computed tomography (CT) scan images showing the results of the chest wall reconstruction using bone cement. (**H**) The gap between cement and the mediastinal structures (white arrow)

**Fig. 2:**
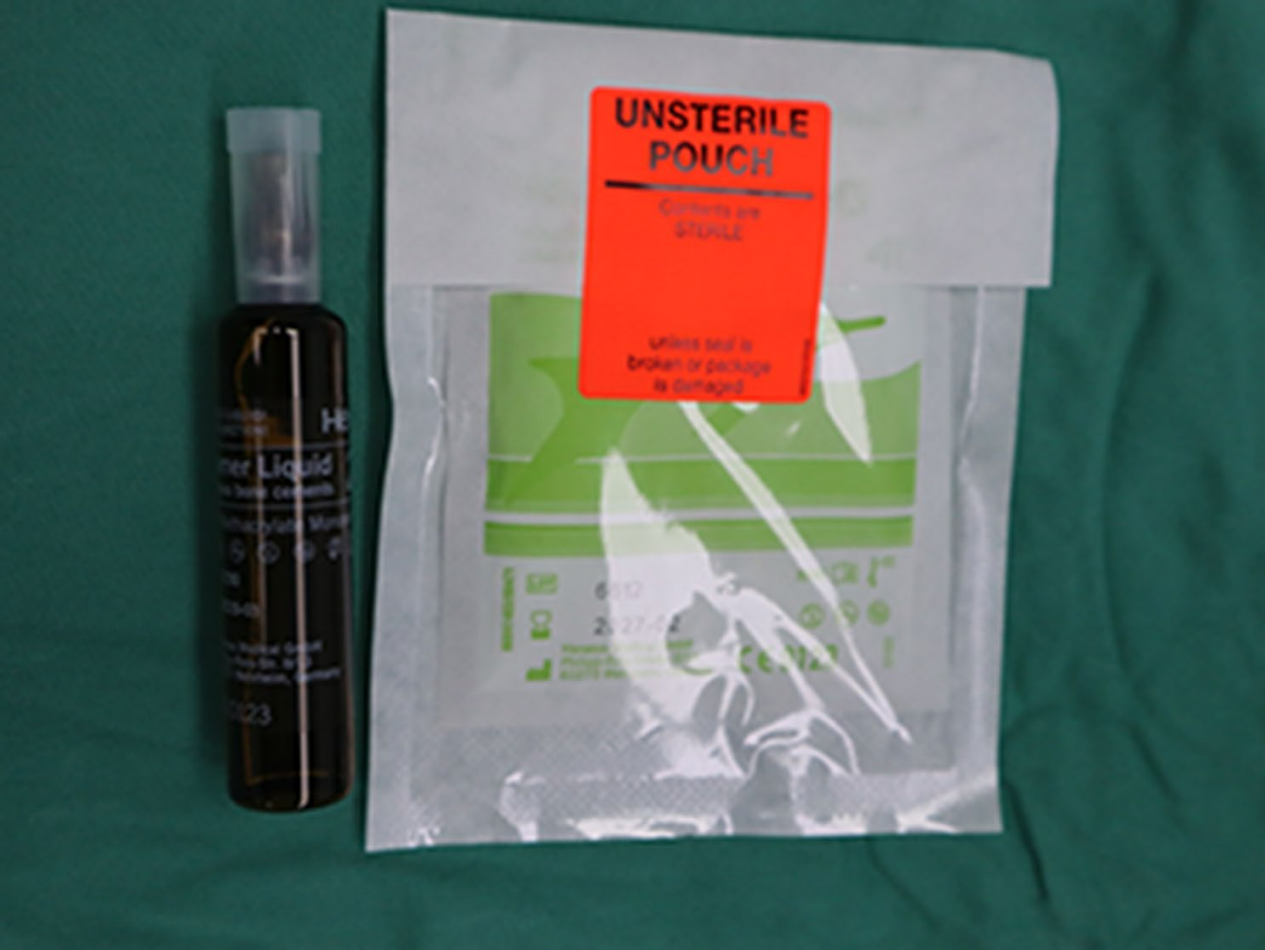
PALACOS MV®+G bone cement is obtained by mixing a poly power component with a liquid monomer component

**Fig. 3:**
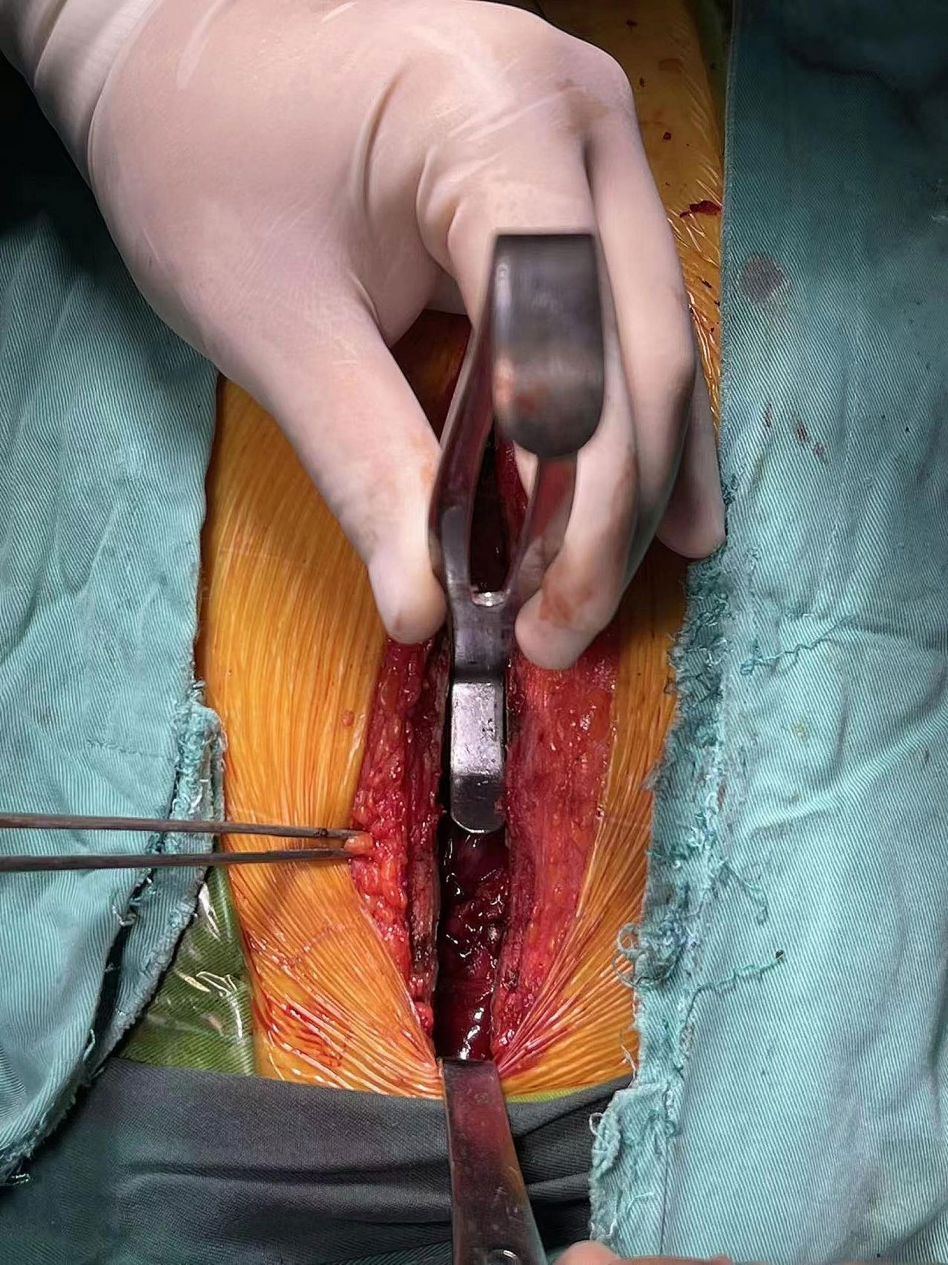
The thyroid retractor was used to prevent the bone cement from sticking to the mediastinal structures

**Fig. 4:**
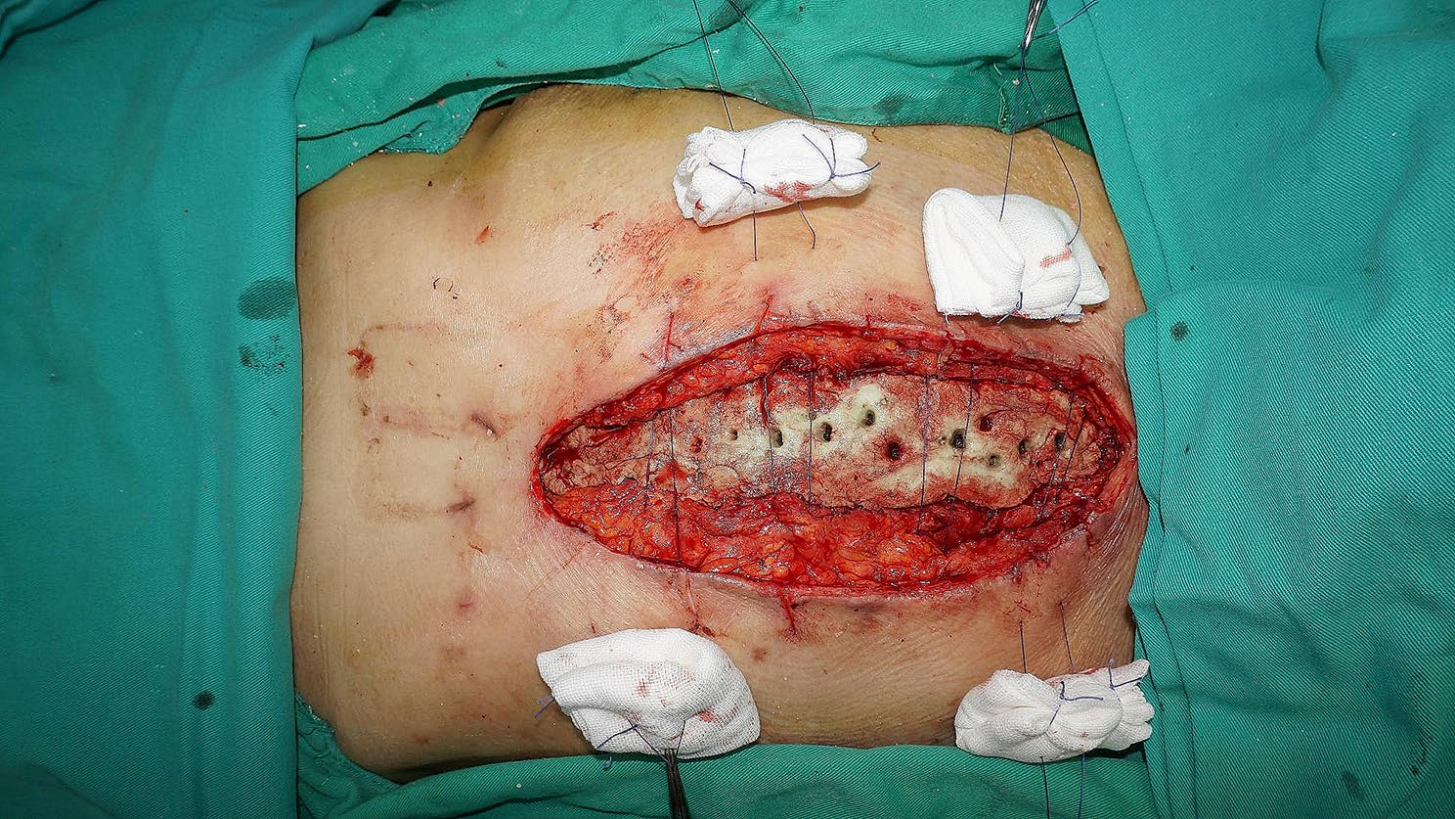
Detachment of the bilateral pectoralis muscle from the chest wall and from the subcutaneous layer. The pectoralis muscle flap and subcutaneous tissues were sewn with muscle flap on the other side by 1 − 0 monofilament absorbable suture

Deep sternal wound infection has been classified as a complex wound, and its treatment is a challenge for the cardiothoracic surgeon. The multidisciplinary team at our center includes cardiothoracic surgeon, cardiac anesthesiologist, chronic wound surgeon, pharmacist, and nutritionist. All procedures were performed under general anesthesia. Arterial blood pressure monitoring and deep venous catheterization were required. The patients were operated on by a team of cardiothoracic and chronic wound surgeon. After microbiological specimens were collected, all infected necrotic tissues and bones were removed until healthy solid bone with bleeding margins was found (Fig. 1C, D). In relation to the local bacterial ecology, four patients received vancomycin-loaded bone cement and one for gentamicin-loaded bone cement. Our ALBC protocol was to confect by combining a 40 g bag of cement (PALACOS MV®+G bone cement, Heraeus, Heraeus Medical GmbH, Wehrheim, Germany) with 2 g of vancomycin or 3.2 g gentamicin powder according to antimicrobial susceptibility test result (Table 2). PALACOS MVⓇ+G bone cement is a radiopaque, quick-setting bone cement with the addition of gentamicin as an antibiotic and exhibits low initial viscosity. It is obtained by mixing a poly power component with a liquid monomer component (Fig. 2). When the powder component mixed fully with the antibiotic, liquid monomer component was added to the mixture. Bone cement was stirred manually, put into the gap between the sternal halves and covered the sternal defect to fix the thorax, during which the heart surface was protected with thyroid retractor to prevent the bone cement from sticking to the mediastinal structures (Fig. 3). However, the process of polymerization of the cement is an exothermic reaction with temperatures up to 60–80°C. ALBC was covered with ice-wet cotton to reduce the local temperature from burning the tissue. Within minutes, the bone-like adhesive adds mechanical strength and stabilizes the reapproximated sternal (Fig. 1E). The wounds were then cleaned and rinsed with normal saline water for 3 times after ALBC implantation. Subsequently, the bilateral pectoralis major muscle flaps and subcutaneous tissue were mobilized from the chest wall (Figs. 1F and 4). The pectoralis muscle flap and subcutaneous tissues were sewn with muscle flap on the other side by 1–0 VICRYL sutures for tension-free (Figs. 1F and 4). At last, the entire layer of the skin and the subcutaneous tissue were sutured intermittently. We leave a flat drainage tube (Disposable negative pressure drainage pipeline, AY-Y18-Q400, Ai yuan medical technology, Jiangsu, China) between pectoralis muscle flaps and the ALBC as well as a grooved drainage tube (Disposable negative pressure drainage pipeline, AY-Y22-Q400, Ai yuan medical technology, Jiangsu, China) below the ALBC, both remained until drainage is less than 10 ml per day for 3days. If there is no drainage tube behind the bone cement, the holes should be made on the surface of ALBC for drainage (Fig. 4).

**Table 2:** Patient sternal wound characteristics

Variable	1#	2#	3#	4#	5#
Symptoms of DSWI	Chest pain Fever Purulent discharge Sternal instability	Chest pain Purulent discharge Sternal instability	Fever Sternal instability Abundant purulent discharge	Chest pain Sternal instability Purulent discharge	Fever Sternal instability Purulent discharge
Initial tissue cultures	Enterococcus faecalis	Enterobacter cloacae	Staphylococcus epidermidis	Staphylococcus aureus	Staphylococcus epidermidis
Blood culture	Negative	Negative	Staphylococcus epidermidis	Negative	Negative
Tracheotomy	No	No	Yes	No	No
Number of debridement before implantation surgery	0	2	0	0	0
Use of VSD therapy	No	Yes	No	No	No
Intravenous antibiotic	Linezolid	Piperacillin/Tazobactam	Linezolid	Cefatriaxone	Linezolid
Interval from cardiac surgery to symptom development of DSWI (days)	12	20	30	25	28
Interval from the diagnosis of infection to reconstruction surgery (days)	3	2	4	2	3
Reconstruction surgery antibiotic loaded /dose(g)	vancomycin/2.0	Gentamicin/0.32	vancomycin/2.0	vancomycin/2.0	vancomycin/2.0
ICU (days)	2.0	–	6.0	–	–
Albumin beforet-Implantation surgery (g/L)	31.6	32.4	25.6	33.4	30.5
Albumin seven days post-Implantation surgery (g/L)	38.2	36.6	32.8	39.6	36.1
Duration of systemic antibiotic(days)	16	12	23	10	16
Length of drainage tube use(days)	30	31	26	29	36
Follow-up duration after implantation surgery (months)	25	20	6	12	35

*Abbreviations* DSWI, Deep sternal wound infection; ICU, Intensive care unit; VSD, Vacuum sealing drainage

### Vancomycin concentration determination

Serum and drain fluid samples were collected at 1d, 3d, 5d, 7d, 9d, 11d, 13d, 15d, postoperatively. Drain fluid samples were from the flat drainage tube, which is between pectoralis muscle flaps and the ALBC.

### Post-operative care/care after cardiac surgery

Individuals’ nutritional status influences both their susceptibility to infection and their response to infection in terms of clinical outcome. Nutritional support through an energy-and carbohydrate-rich diet was recommended. According to the anticoagulation requirements after cardiac surgery, oral warfarin, warfarin with aspirin or aspirin with tigrillo was taken, and the International normalized ratio (INR) was maintained at 1.8–2.5. The systemic antibiotics were continued in accordance with bacterial sensitivity until inflammatory mediators’ levels were satisfactory (CRP < 5.0 mg/L and ESR < 15 mm/h).

### Wound management

**Fig. 5:**
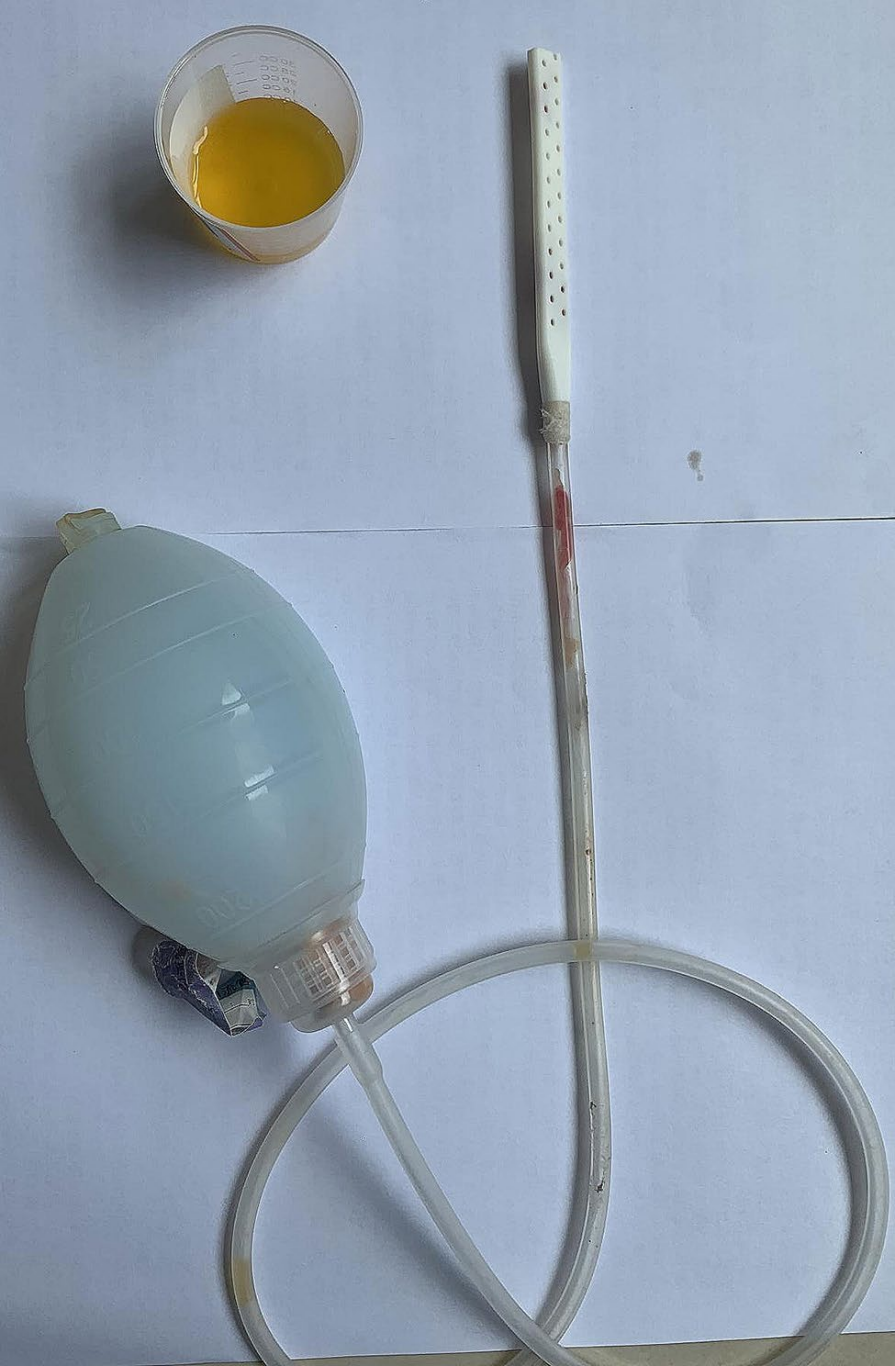
The drainage tube was relieved for only a little yellow fluid being drained off when volume was less than 10 mL/24 h for three days

Wound management was performed using only sterile gauze. The wound was observed daily for redness, pain, drainage parameters, bleeding, subcutaneous effusion and skin flap necrosis. If there were no wound problems, tension-free sutures were removed 2 weeks later. Skin sutures were removed 3 weeks later. When to pull out the drainage tube? The drainage fluid was light yellow, and the drainage volume was less than 10 mL/24 h for three days (Fig. 5). Elastic bandage fixated the chest wall for 3 months.

### Statistical analyses

Continuous variables were expressed as mean ± standard deviation. A paired t-test was performed to evaluate the changes in vancomycin concentrations values. A P value of ≤ 0.05 was considered statistically significant.

## Results

### Surgical outcomes

The results of bacterial culture of secretions taken from the wounds of the patients before the operation: Patient 3 and Patient 5 were positive for *Staphylococcus epidermidis*, Patient 4 was positive for *Staphylococcus aureus*, Patient 1 was positive for *Enterococcus faecalis*, and Patient 2 was positive for *Enterobacter cloacae*. Four patients were treated with vancomycin-loaded bone cement and one patient with gentamicin-loaded bone cement according to drug sensitivity (Table 2). For all patients, one-stage healing was achieved within about 3 weeks after surgery. No patient underwent any invasive surgical procedures except for the initial wound opening, or incision and drainage. The median length of drainage tube use was 30.4 (26–36) days and median hospital stay was 24.0 (18–30) days. There were no perioperative mortalities, no postoperative complications, 100% wound healing. All patients were discharged without sternal infection symptoms. Patient 3 developed pulmonary infection and respiratory failure during the 6-month follow-up due to tracheotomy. The mean follow-up was 19.6 months (range 6–35 months). All patients had stable chest walls with no further sternal instability, no recurrent dehiscence or wound infections and there were no hepatic or renal insufficiency during the follow-up.

### Vancomycin concentrations

**Fig. 6:**
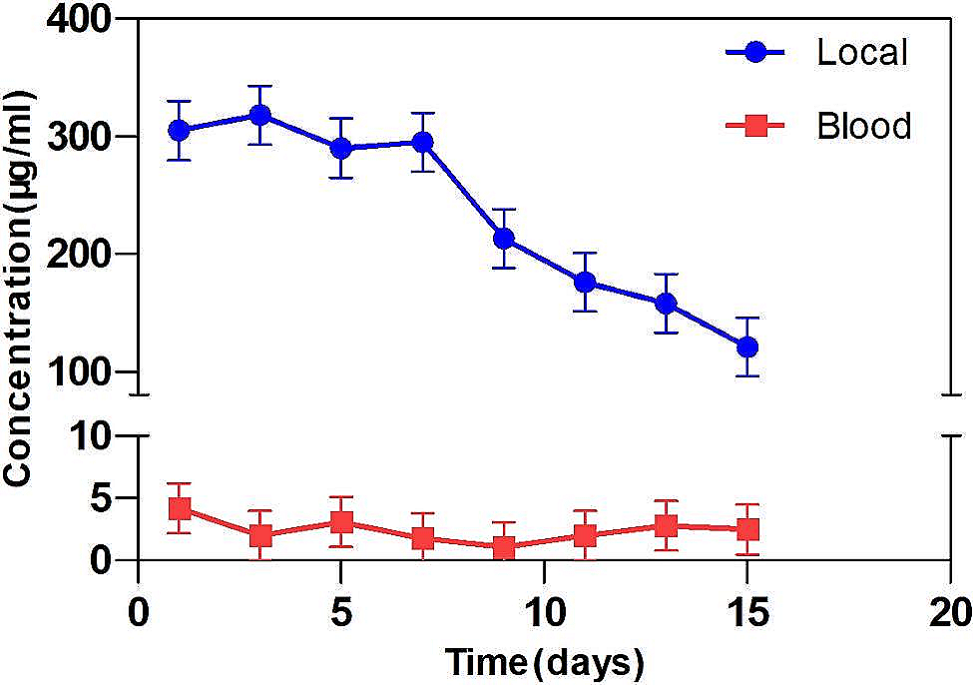
Release of vancomycin in local tissue and blood over the course of days from bone cement

Local and serum concentrations at every time point were summarized in (Fig. 6).

After implantation of vancomycin-loaded bone cement, local vancomycin showed a significant therapeutic level of at least 30 times (305 ± 42 µg/mL) than the minimal inhibitory concentration (10–15 µg/mL) 24 h post-surgery. This high concentration of local vancomycin lasted for at least 2 weeks, ensuring the eradication of bacteria. The minimum local drug concentration was (121 ± 33 µg/mL) within 2 weeks. Serum concentrations of vancomycin was sub-toxic (< 10 µg/ml ) in the all patients. These differences were highly significant (*P*<0.01).

## Discussion

DSWI is a rare but potentially devastating complication of median sternotomy after cardiac surgery [24, 25]. Risk factors for sternotomy wound complications include osteoporosis, obesity, COPD, diabetes, corticosteroid use, reoperation, use of internal mammary arteries, poor nutritional status, prolonged surgery, persistent postoperative coughing, inappropriate timing of prophylactic antibiotic administration, inadvertent paramedian sternotomy and kidney disease [26–29]. Several of these risk factors are commonly coexistent in cardiac surgery populations. The causative factors of DSWI in this study included: (I) Limited blood supply to the sternal due to internal mammary artery harvest; (II) sternal fracture caused by wire and fixation device; (III) Severe sternal osteoporosis; (IV) ventilator-associated blood-borne infections; and (V) the large displacement of the sternal.

Treatment of the DSWI has improved significantly over the past half century. The optimal treatment strategy for DSWI is still under debate. Now, vacuum-assisted therapy combined with turnover flaps remains the current standard therapeutic strategies [30, 31]. Options for sternal reconstruction typically include the pectoralis major, omentum, rectus abdominis, or a combination of flaps [26, 32]. While many techniques for sternal reconstruction have been tried, each has its limitations. Among the greatest limitations of all strategies for treating DSWI are the inability to quickly improve sternal stability, provide reliable mechanical support to achieve fast physical function recovery and avoid potential damage to heart and great vessels.

Our treatment strategy for DSWI was built on four concepts: (1) A multidisciplinary collaboration to develop and implement surgical treatment plans, (2) early debridement and sternal reconstruction, (3) suitable material for sternal reconstruction, (4) Peri-operative management: improve nutritional status, stable internal environment, optimized anti-infective therapy. DSWI is defined as a bone-related infection and the two most important points to be dealt with were sternal instability and infection [33]. Sternal instability can cause a life-threatening problem with postoperative respiratory failure, chronic pain, deformity of the thoracic wall. So the therapeutic concept should consider mechanical and functional aspects. Bone cement can provide sufficient material to fill the sternal defect and stabilize the sternal, with no pathological movement of the chest wall during breathing or activity, no signs of respiratory insufficiency and painful rubbing and clicking of bony edges. After implantation, the setting PMMA undergoes polymerization; the exothermic reaction heating the surrounding bone up to 70°C, which can cause tissue necrosis of the nociceptive nerve terminals, thus numbing pain.

Given significant morbidity and mortality, patients with suspected DSWI should be immediately started on broad-spectrum antibiotics with eventual narrowing of coverage based on wound culture [34, 35]. Systemic antibiotics, which are commonly used to treat DSWI or sternal osteomyelitis, may not be sufficiently effective to avoid deep infection because of impaired blood circulation and low antibiotic concentrations at the infection site, especially for internal mammary artery harvesting [36, 37]. Simultaneously, it can lead to systemic toxicity and drive bacterial antibiotic resistance. Antibiotic-loaded bone cement is used in a variety of applications, such as the temporary spacer during a two-stage revision surgery for total hip arthroplasty, the cement interfaced between the implant and native tissue in arthroplasty load-bearing applications, and the antibiotic beads for treating osteomyelitis [38]. The antibiotic release offered by this type of bone cement is used as a local delivery system, with high localized concentrations, did not enter the bloodstream in significant amounts [39]. The aminoglycosides gentamicin and tobramycin are the most common antibiotics commercially preloaded into bone cement, while vancomycin is the most common antibiotic added to bone cement [40]. Staphylococcus was the most common pathogenic bacterium in DSWI patients [41, 42]. The problem of nephrotoxicity is an important factor in patients who have undergone cardiac surgery procedures, where the incidence can reach 30% [43]. However, vancomycin can manifest obvious systemic toxicity toward the human body, from which nephrotoxicity is the most common effect [44]. Therefore, it is necessary to detect vancomycin serum concentration. In this study, we selected vancomycin-loaded bone cement for four patients with Gram-positive bacterial infection according to drug sensitivity, systemic antibiotic was started postoperative until the biochemical markers of inflammation returned to the normal ranges. As described here, vancomycin-loaded bone cement delivered high concentrations of vancomycin locally within the wound with low serum concentration levels (Fig. 6), preventing the systemic adverse effects and no hepatic or renal toxicity were notable.

Partial sternal and soft tissue were lost due to infection and radical debridement. Meanwhile, bone cement was inserted between the sternal halves. Excessive skin tension may occur for the closure of sternal infection wound. Tension-free wound management was chosen to close the wound with bilateral-pectoralis major muscle advancement flap in order to cover the surface of bone cement and prevent wound dehiscence. Suture hypersensitivity refers to an inflammatory foreign body reaction resulting from an exaggerated immunologic response triggered by the presence of a suture material, which serves as an external antigen [45]. By applying this suture method, no foreign body remained after the stitches were removed, thus reducing the risk of recurrence of infection.

As DSWI is complex and hard-to-heal, the management of this wound infection continues to be challenging. The reconstruction of DSWI requires the cooperation of a multidisciplinary team. Cardiothoracic surgeons and plastic surgeons should learn from each other, so as to wait for better treatment effects. In addition, abundant preoperative preparation and strict postoperative management are also critical to success. To the best of our knowledge, this study is the first to apply such a technique for the treatment of DSWI after cardiac surgery. Our procedure is simple, does not require advanced skills. We believe that ALBC combined with BPMMFs has the potential to become the first-line reconstructive procedure for DSWI.

## Limitations

The primary limitation of this study is its small sample size based on its period study. Our work is a single-center, observational, and retrospective data analysis study, further research with larger sample sizes is warranted. However, considering the rarity of DSWI and the subsequent ALBC with BPMMFs reconstruction, randomized study design remains challenging. The study focused on showing the surgical method using ALBC combined with BPMMFs, the relatively short follow-up to its long-term efficacy need time to verify. It is not sure whether ALBC should be removed and when to remove, or whether it will cause foreign body reaction, which needs further clinical research.

## Conclusions

In the present report, we present a retrospective review of 5 patients with deep sternal wound infection successfully treated with ALBC fixation technique combined with BPMMFs tension-free management. Sternal reconstruction by ALBC successfully restored the chest wall rigidity, preserved respiratory function and protected the mediastinal organs. Additionally, ALBC acted as local antibiotic delivery system, which was not dependent on vascularization of target tissue that maintained high local antibiotic concentrations over a prolonged period of time with minimal systemic absorption. BPMMFs tension-free management does not only provide sufficient tissue to cover the ALBC but can also leave no dead space and reduce foreign body reaction. Based on our satisfactory experience, it is promising to be used in other centres for treating patients with DSWI in an excellent clinical response.

## Data Availability

The datasets generated and analyzed during the current study are not published due to the use of internal records of patient data and established privacy policies, but will be available from the corresponding author upon reasonable request.

## Abbreviations

ALBC: Antibiotic-loaded bone cement
BPMMF: Bilateral pectoralis major muscle flaps
DSWI: Deep sternal wound infection
CRP: C-reactive protein
ESR: Erythrocyte sedimentation rate
VSD: Vacuum sealing drainage
COPD: Chronic obstructive pulmonary disease
